# A unique posterior nutcracker syndrome combined with Wilkie syndrome: A singular case

**DOI:** 10.1016/j.radcr.2024.05.050

**Published:** 2024-06-07

**Authors:** Dario Milazzo, Francesco Tiralongo, Renato Farina, Pietro Valerio Foti, Corrado Ini', Monica Palermo, Mariapaola Tiralongo, Davide Giuseppe Castiglione, Emanuele David, Stefano Palmucci, Antonio Basile

**Affiliations:** a Radiology Unit 1, Department of Medical Surgical Sciences and Advanced Technologies “GF Ingrassia”, University Hospital Policlinico “G. Rodolico-San Marco”, University of Catania, 95123 Catania, Italy; b Radiology Unit 1, University Hospital Policlinico “G. Rodolico-San Marco”, 95123 Catania, Italy; c Department of Clinical and Experimental Medicine, University of Catania, Catania, Italy; d UOSD I.P.T.R.A., Department of Medical Surgical Sciences and Advanced Technologies “GF Ingrassia”, University of Catania, University Hospital Policlinico “G. Rodolico-San Marco”, Catania, Italy

**Keywords:** Posterior nutcracker syndrome, Superior mesenteric artery syndrome, Left venal rein, Iliac artery, Computed tomography, Case report

## Abstract

Left renal vein variants are not commonly observed in the general population. Usually, the renal vein runs in front of the aorta before entering the inferior vena cava, while the most common variants include the presence of a circumaortic or retroaortic renal vein. However, when present, left venal rein variants are important to recognize due to their potential clinical and surgical relevance. In this regard, CE-CT is an instrument with high sensitivity and specificity in detecting vascular anomalies and can certainly help diagnose. In this article, we present a unique case of a left venal rein compressed between the left iliac artery and vertebral bodies associated with the presence of a superior mesenteric artery Syndrome, another rare entity that occurs when the duodenum is compressed between the aorta and the superior mesenteric artery.

## Introduction

Because of the increased use of advanced imaging techniques, such as contrast-enhanced computed tomography (CE-CT), the occasional finding of abnormalities of the inferior vena cava and its tributaries has become more frequent: the retroaortic left venal rein is one of these anomalies, but in most cases, it remains asymptomatic [1]. Nevertheless, the latter may be compressed between the aorta and vertebral bodies in some cases, identifying the so-called posterior nutcracker phenomenon. This occurrence may be accompanied by specific symptoms, configuring posterior nutcracker Syndrome [2].

We report here a case presenting both compression of a retroaortic left venal rein and superior mesenteric artery syndrome.

## Case report

We report a case of a 55-year-old Caucasian male admitted to our emergency department for an acute onset of nonspecific meso/hypogastric abdominal pain with episodes of biliary vomiting.

His past medical history was characterized by intermittent abdominal pain in the left flank region and reported weight loss within the past 6 months. On physical examination, mild abdominal distension was noted, with pain on palpation mainly in the upper abdominal quadrants (especially in the epigastrium). He weighed 63 Kg and was 176 cm tall, giving him a body mass index (BMI) of 20.3 Kg/m^2^.

However, laboratory tests were unrevealing.

Nevertheless, given the high clinical suspicion of small bowel obstruction, an urgent abdominal CE-CT examination was performed using a multi-detector computed tomography (Revolution EVO, GE Healthcare, Chicago, IL, USA).

**Fig. 1:**
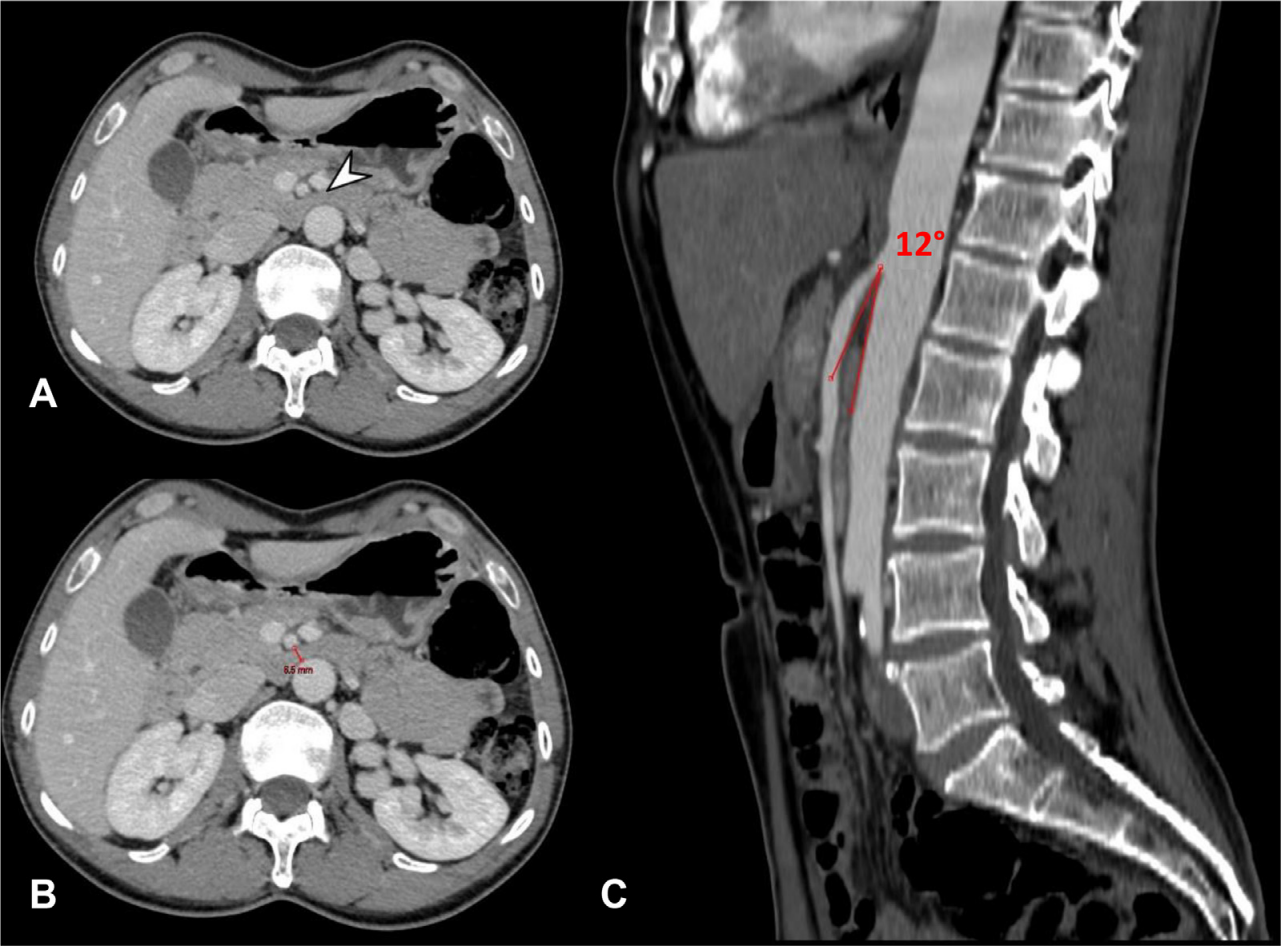
(A-C) Superior mesenteric artery syndrome. The SMA syndrome (also known as Wilkie's syndrome) is diagnosed radiologically when, in the presence of symptoms with the characteristics of a high intestinal occlusion, the presence of an angle between the SMA and the aorta <22° or when the aortomesenteric distance is <8 mm. Axial CE-CT images of our case (A,B), obtained in portal venous phase, demonstrate the presence of duodenal compression (arrowhead in A) and a maximum aortomesenteric distance of 6.5 mm (red segment in B). In addition, sagittal CE-CT image (C) shows the presence of an aortomesenteric angle of approximately 12° (red angle in C).

CT scan did not reveal any definitive findings indicative of intestinal obstruction. However, a finding was identified which could potentially contribute to the patient's clinical presentation. The CT scan demonstrated compression of the third portion of the duodenum between the superior mesenteric artery (SMA) and the aorta, consistent with superior mesenteric artery syndrome, also known as Wilkie syndrome. In particular, the angle between the vessels measured 12° degrees (Fig. 1).

Additionally, CE-CT causally revealed a left renal vein (LVR) with an anomalous retro-aortic course before draining into the inferior vena cava: the LVR was found to be so compressed between the left common iliac artery and fourth lumbar vertebra's body. A distance of 3.4 mm between the common iliac artery and the anterior aspect of the fourth lumbar vertebral body (L4) was measured.

**Fig. 2:**
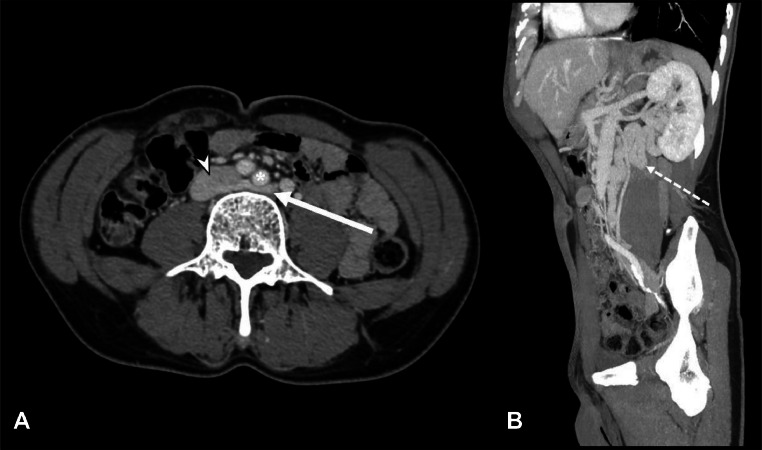
(A,B) Retroaortic left venal rein compression. Axial CE-CT in venous phase (A) shows the retroaortic renal vein (arrow) compressed by the left iliac artery (asterisk), before flowing into the inferior vena cava (arrowhead). Furthermore, a parasagittal section obtained by MIP reconstruction (B) shows considerable ectasia of the renal vein tract upstream of the compression (dotted arrow in B).

As a result, the LVR was remarkably dilatated throughout its course upstream of the obstruction: on CT multiplanar reconstructions (MPR), it was possible to observe the pathological serpiginous vein dilatation (Fig. 2). These findings were consistent with posterior nutcracker syndrome (NCS): however, due to its anatomical characteristics, this was a unique finding.

An intraductal papillary mucinous neoplasms branch duct type was found as a collateral finding.

**Fig. 3:**
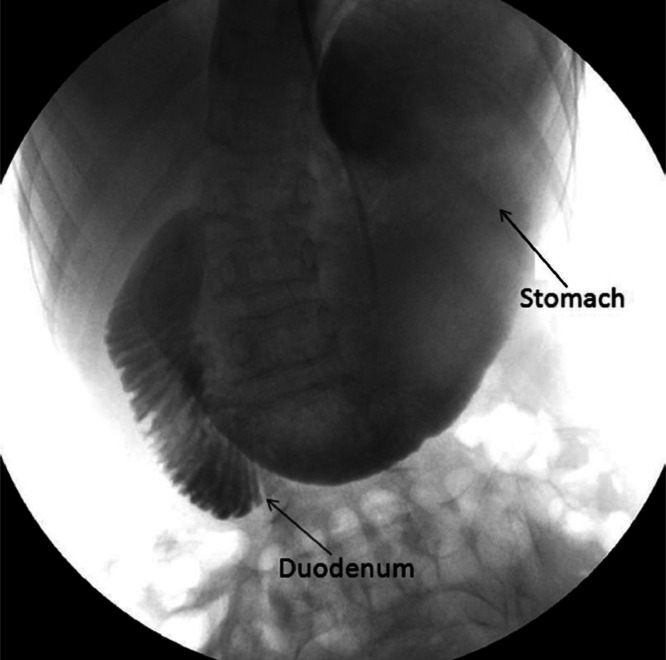
Gastroduodenal fluoroscopy. The fluoroscopy with iodinated contrast medium shows gastro-duodenal dilatation up to the third portion of the duodenum, which was previously found compressed by the superior mesenteric artery in CT with contrast medium. The last duodenal portion exhibits no contrast, suggesting that the contents’ passage is obstructed.

After a few hours, the patient's symptoms decreased significantly, and he was discharged and sent for a gastroduodenal fluoroscopy and an esophagogastroduodenoscopy for further investigations. The fluoroscopy with iodinated contrast medium confirmed this diagnosis, showing gastro-duodenal dilatation up to the third portion of the duodenum, which was compressed by the SMA and collapsed immediately after crossing it (Fig. 3).

## Discussion

In normal anatomy, each kidney drains to the inferior vena cava (IVC) through a single vein. After receiving the left adrenal and gonadal veins, thus identifying a “proximal” and a “distal” segment [3], the LVR runs horizontally in front of the aorta and ends in IVC, commonly at the level of the first 2 lumbar vertebrae [4]. Anomalies in embryological development can, however, lead to the presence of variants: usually, a pair of renal veins, 1 ventral, and 1 dorsal, provide venous drainage during fetal life, thus forming a “collar” around the aorta itself.

Then, during development, the dorsal vein is expected to disappear. Hence the possibility of the 2 most common variants, the “circumaortic venous ring”, when both fetal veins persist, and the “retroaortic left venal rein” (RLVR), when the ventral vein atrophies [5].

**Fig. 4:**
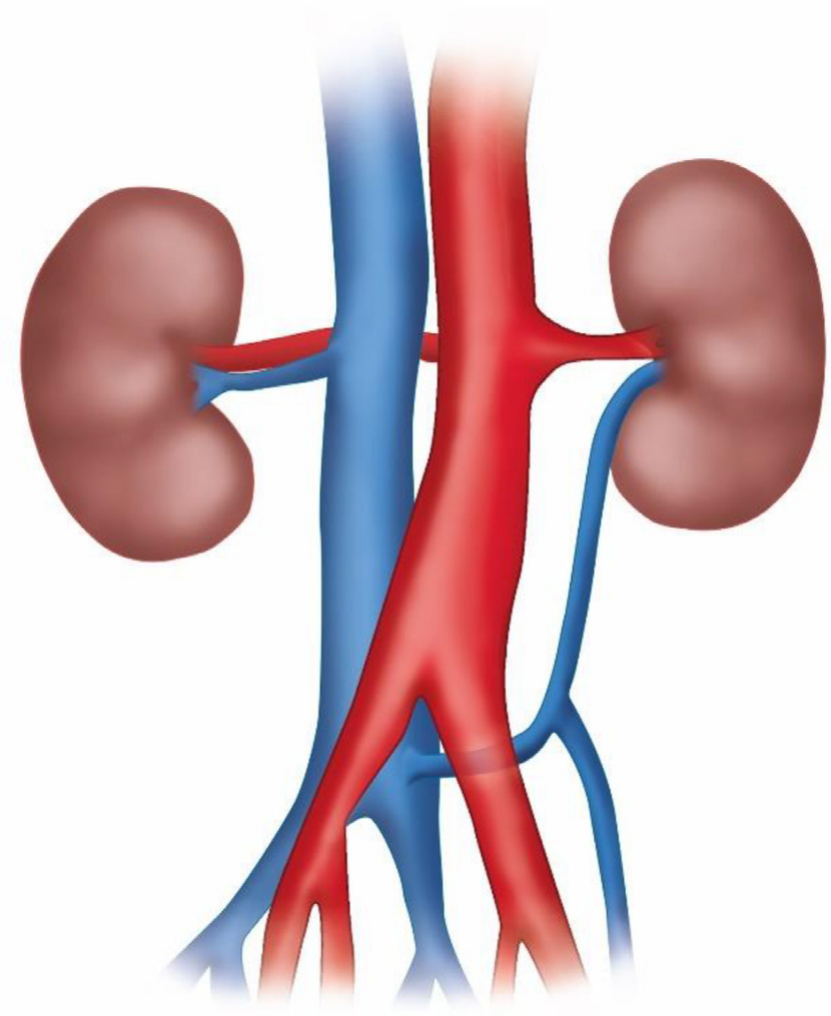
Graphic illustration of left renal vein with its retro-iliac artery course. The left renal vein originates from the renal hilum, running down and medially behind the left iliac artery, ending on the lateral side of the inferior vena cava. This is a unique type of posterior nutcracker syndrome.

Although rare and usually asymptomatic (can occasionally cause flank pain or hematuria), these variants are of considerable surgical importance if present: radiological diagnosis, therefore, plays a central role. Indeed, it is essential to know LVR anatomy to prevent complications in many surgical procedures like nephrectomy, kidney transplantation, treatment of abdominal aortic ectasia, and retroperitoneal lymphadenectomy [3]. Although many classifications of left venal rein anomalies have been proposed [6], the most currently used 1 provides for 4 types of abnormality. In particular, in type I and type II, a single RLVR joins the IVC in an orthotopic position and L4-L5 level, respectively, while the circumaortic venous ring is classified as type III; in type IV, the RLVR drains into the left common iliac vein [1]. In our case, however, the RLVR, even if present, reached the vena cava at L4 level after a course posterior to the left common iliac artery (CIA) (Fig. 4). To the best of our knowledge, there is no evidence in previous literature of a similar case.

As mentioned above, the RLVR was also compressed between the left CIA and L4 vertebra's body. Compression of the left renal vein can cause NCS: in fact, any altered venous return can be reflected upstream in the left gonadal vein, resulting in venous ectasia, and slowing of the flow of both veins. This syndrome may manifest itself with pelvic varices, intermittent hematuria, up to gonadal or spermatic reflux, or can be asymptomatic (so called “nutcracker phenomenon”) [2].

Two types of NCS have been described: an anterior NCS, where the LVR is compressed by the aorta and SMA, and a posterior NCS, where RLVR is entrapped between the aorta (or iliac arteries) and lumbar vertebrae, as in the case we report here [7]. Our patient's symptoms could be related to these anomalies, identifying a posterior nutcracker syndrome incidentally discovered thanks to CE-CT. In this regard, CE-CT has high diagnostic accuracy in vessel studies, allowing the recognition of LVR anomalies and imaging findings of nutcracker syndrome [5].

**Fig. 5:**
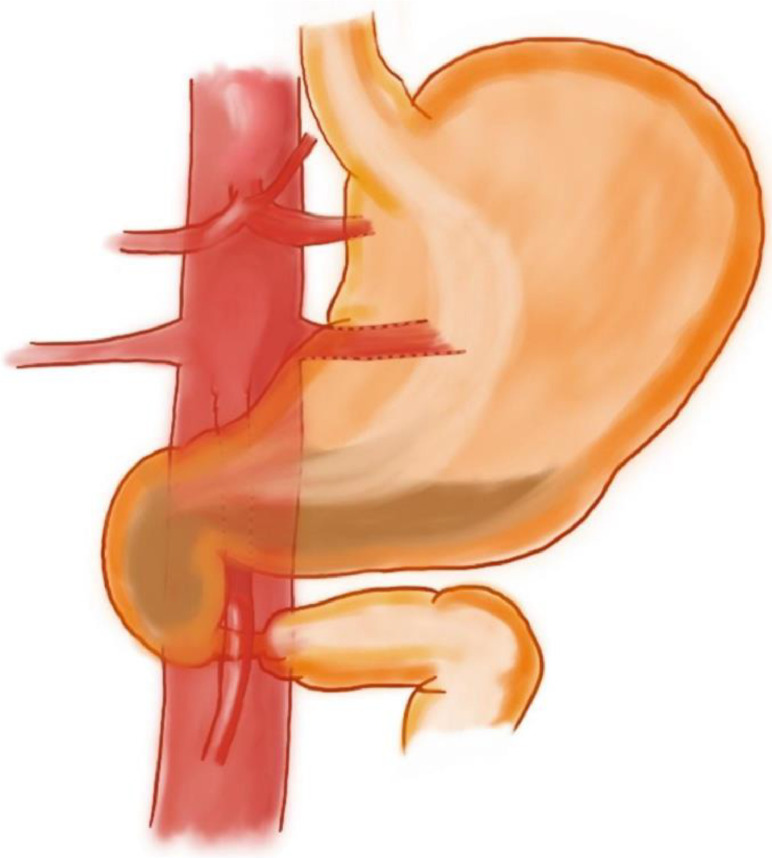
Graphic illustration of superior mesenteric artery syndrome: It is possible to observe the duodenum compressed between the abdominal aorta and the overlying superior mesenteric artery, with relative dilatation of the duodenum upstream the compression. This condition is called superior mesenteric artery (or Wilkie's) syndrome.

Our patient's subocclusion symptoms may instead be explained by the simultaneous presence of a SMA (or Wilkie's) syndrome, namely the duodenal compression between the aorta and the SMA, as mentioned above (Fig. 5). In fact, this rare condition, when manifested acutely, is characterized by signs and symptoms of duodenal obstruction [8]. Normally, the duodenum is surrounded by adipose tissue, which prevents extrinsic compression. However, when this is absent due to pathological conditions, such as severe catabolic states, or paraphysiological conditions, such as significant weight loss or anatomical changes, adipose tissue's function may be compromised. These circumstances can lead to a marked reduction in the angle between the aorta and the SMA, with the latter compressing the duodenum; in severe cases this can cause a small bowel obstruction. Additionally, Wilkie's syndrome can be associated with nutcracker syndrome. This combination is rarely described in the literature, and it is usually the anterior nutcracker syndrome that occurs in association with SMA syndrome, as they share the same aetiopathogenetic basis (i.e. compression of the left renal vein and duodenum, respectively, within the aortomesenteric angle) [9,10]. For these reasons, the co-occurrence of SMA and posterior nutcracker syndrome is even more unusual [11].

In CE-CT, the diagnosis of SMA syndrome is made by measuring the angle between the SMA and the aorta, known as the aortomesenteric angle, considered pathognomonic of SMA syndrome when inferior to 22°. Alternatively, the aortomesenteric distance can be measured: if it is <8 mm, it is diagnostic of this syndrome [8]. With the help of CT MPRs, especially in sagittal view, it was possible to diagnose this rare syndrome and to explain the patient's symptoms (Fig. 1).

These elements, taken together, account for the uniqueness of the case described here, where a unique type of LVR/Nutcracker phenomenon coexists with another uncommon condition such as the SMA syndrome.

Finally, it is important to highlight the diagnostic potential offered by CT in recognizing rare vascular disorders, including vascular compression syndromes [12].

## Conclusion

Although rare and often encountered occasionally, compression syndromes may be symptomatic but misdiagnosed. A deeper understanding of their many possible presentations is therefore important and now possible thanks to technological advances in radiology.

## Patient consent

Written informed consent for this case report was obtained from the patient.
